# Pulsating Drought and Insect Herbivory Cause Differential Effects on Soybean ( *Glycine max* ) Genotypes That Vary in Canopy Wilting Speed

**DOI:** 10.1002/pei3.70028

**Published:** 2025-01-30

**Authors:** Jessica Ayala, Manish Gautam, Adriana Peissel, Justin George, Rupesh Kariyat

**Affiliations:** ^1^ Department of Entomology & Plant Pathology University of Arkansas Fayetteville Arkansas USA; ^2^ Southern Insect Management Research Unit USDA‐ARS Stoneville Mississippi USA

**Keywords:** canopy wilting, drought, fall armyworm, herbivory, soybean looper, water use efficiency

## Abstract

As a result of climate change, global temperatures are increasing, and water scarcity is on the rise. Soybean [ *Glycine max* (*L.*) Merr] is one of the most important crops in the world due to its importance as food and feed. One of the major limiting factors for soybean production is drought, which can cause up to 80% reduction in yield. Therefore, growers and plant breeders are turning to soybean accessions that demonstrate better water use efficiency (WUE). However, in addition to drought, insect herbivory by soybean looper (*Chrysodeixis includens,* SBL) and fall armyworm ( *Spodoptera frugiperda* , FAW) can also reduce soybean yield by feeding on foliar and floral organs. Using soybean accessions that differ in their wilting speed, we examined the relationship between physiological traits associated with WUE, and how they affect both herbivore and host plant growth and development. Results showed that both fast‐ and slow‐wilting genotypes displayed strong overcompensation in terms of growth and development, but slow‐wilting genotypes produced higher‐quality pods and seeds. Regardless of treatment effects, FAW fed at a significantly higher rate than SBL despite being less specialized to feed on soybeans. While fast‐wilting plants produced more pods than slow‐wilting plants regardless of treatment, slow‐wilting plants produced heavier pods with larger and heavier seeds. Collectively, we show that despite fast‐wilting plants overcompensating in pod production and growth traits, slow‐wilting plants may still be better fit through seed functions.

## Graphical abstract

Soybean plants exhibit overcompensation when exposed to both insect and drought stress. There are strong genotypic effects within genotypes that vary in wilting speeds. Fast‐wilting plants produced more seeds, but slow‐wilting plants produced higher‐quality seeds.

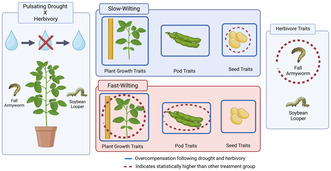

## 1 Introduction

Agricultural crops that are largely grown in tropical and subtropical regions are facing record breaking high temperatures and extended periods of drought in recent years, possibly due to climate change (Walsh et al. 2020; FAO 2024). Soybean [ *Glycine max* (*L*.) *Merr*.] is one of the most important agricultural crops in the world with various roles in the agricultural sector. It is highly valued due to its nutritional value in the form of protein, oils, and micronutrients (Choudhary and Tran 2011; He and Chen 2013). Soybean serves as a major source of protein for both humans and animals, contributing to more than 70% of protein consumption of each meal (Fried, Narayanan, and Fallen 2019; FAO 2024). A large portion of soybean production contributes to animal feed, and about 60% of oil seed in the world is soybean oil, thus elevating the importance of soybeans in global agriculture (Fried, Narayanan, and Fallen 2019).

Drought is one of the most detrimental abiotic stressors for soybean and can cause over 50% yield loss (Wei et al. 2018). Soybeans are water intensive and require moist conditions which can reduce yield in under‐irrigated areas or in arid regions where crops are largely rainfed. Increasing the number of soybean cultivars that manage stress while sustaining high yields and introgression with higher drought resistance and water use efficiency (WUE) traits is vital to maintaining and improving the yield required to meet demands. Needless to say, investigating traits linked with WUE such as canopy wilting speed or canopy temperature is therefore critical to further develop better soybean cultivars. Canopy wilting is a water conservation trait that refers to the speed at which the soybean plants are visibly experiencing drought stress (Ye et al. 2020). Canopy wilting is associated with soil moisture conservation which in turn aids in maintaining increased leaf water content and sustaining lower canopy temperatures (Chamarthi et al. 2021). Soybeans that have slower wilting speeds tend to have better WUE and drought tolerance traits such as a high C13 ratio and low ureide concentration, while soybeans that have faster wilting speeds tend to have fewer WUE traits and are less drought tolerant, have a low C13 ratio and high ureide concentration (Chamarthi et al. 2021). C13 ratio refers to carbon isotope ratio and has been linked to increase in WUE and drought tolerant traits in soybeans (Chamarthi et al. 2023). Ureides are present when a plant is experiencing drought stress and are linked to lower N_2_ fixation in soybeans (Serraj et al. 1999). Canopy wilting phenotype is easy to identify since it is one of the first visual cues growers will notice when their plants are under drought stress (Fletcher, Sinclair, and Allen 2007; King, Purcell, and Brye 2009; Chamarthi et al. 2021). Determining canopy wilting speed requires no additional equipment which is extremely valuable in smaller farming operations or in underdeveloped countries where access to other methods of screening is not feasible (Kunert and Vorster 2020). Taken together, canopy wilting in soybeans is a trait of growing interest to further understand how environmental stressors affect plant phenotype.

In field conditions, plants rarely face a single threat at a time, and in most cases, they are under attack by both abiotic and biotic stressors, either sequentially or in tandem. The soybean looper (*Chrysodeixis includens*, SBL) is one of the most economically important pests of soybeans, causing a significant reduction in yield once defoliation of the plant reaches a threshold of 20%–35% (Kogan and Turnipseed 1987; Moonga and Davis 2016). While SBL has demonstrated pesticide resistance to many pesticides, the increased usage of pesticides in recent years has also caused a significant decline in the population of natural predators, allowing SBL populations to increase unchecked (Temple et al. 2008). In addition, rising global temperatures may exacerbate SBL damage, since herbivore populations increase when exposed to higher temperatures and humid climates (Soares et al. 2021).

Likewise, fall armyworm (*Spodoptera frugiperda*, FAW) is a prolific polyphagous agricultural pest that is highly invasive, posing a growing threat to soybeans (Kenis et al. 2022). It is estimated that FAW herbivory can cause nearly a 50% total yield loss annually in various field crops (Kumela et al. 2019). Conventional methods of pest management against FAW include the use of pesticides to limit the scope of its damage, but current estimates suggest that FAW has acquired resistance against the active ingredients in 47 pesticides (Mota‐Sanchez and Wise 2021). In addition to the financial strain of constant pesticide use, in FAW, they are only effective in early instars (Sisay et al. 2019; Purdue University 2023). Besides human health concerns, these pesticides have been known to affect nontarget arthropods, especially pollinators, causing a significant decline in insect biodiversity as well as a loss of beneficial insects that are critical for agroecosystem functioning (Serrão et al. 2022). While the impact of drought on herbivores of different field crops like rice and corn is well studied, SBL and FAW herbivory in soybeans under water‐limited conditions are not well understood despite being important lepidopteran pests of soybean (Gautam, Shafi, and Kariyat 2024). Also, the interactions of these herbivores with genotypes of varying wilting speed under drought conditions, and any potential trade‐offs are completely unexplored.

To examine this in detail, we used six fast and six slow‐wilting soybean genotypes and two soybean pests; SBL (soybean‐dominant herbivore) and FAW (generalist herbivore) to test their interactive effects under simulated drought (pulsating drought; Ayala et al. 2024; Gautam, Shafi, and Kariyat 2024). Specifically, we asked the following questions: (1) How does variation in canopy wilting speed affect response to drought stress in soybean genotypes? (2) How does drought influence plant–herbivore interactions? (3) How do plant–herbivore interactions differ between a soybean‐dominant herbivore and a generalist herbivore? and (4) What are the consequences/trade‐offs of these effects on soybean fitness? We hypothesized that the combination of abiotic and biotic stress in the form of drought and insect herbivory will be detrimental to the plant's growth and development. We also predicted that the soybean‐dominant herbivore, SBL, would be able to overcome the plant's defenses and perform better when compared to the generalist herbivore, FAW.

## 2 Materials and Methods

### 2.1 Study Systems

#### 2.1.1 Insect Colony

##### 2.1.1.1 FAW Colony

For all experiments, FAW eggs were purchased from a commercial vendor (Frontier Agricultural Services, Calexico, CA, USA). They were hatched at room temperature (21°C–24°C) and then transferred to plastic containers with an artificial diet prior to experimentation (see Ayala et al. 2024 for rearing details).

##### 2.1.1.2 SBL Colony

For all experiments, SBL eggs were purchased from a commercial vendor (Benzon Research Inc., Pennsylvania, USA). Similar to FAW, the SBL eggs were hatched at room temperature (21°C–24°C) and then transferred to plastic containers with an artificial diet prior to experimentation.

#### 2.1.2 Plant Propagation

All soybean genotypes were acquired from the USDA GRIN database. Soybeans were grown in greenhouse at Milo J. Shult Agricultural Research & Extension Center (SAREC), Arkansas Agricultural Experiment Station of the University of Arkansas on a 16:8 light:dark cycle with ~70% humidity conditions in a 28°C–30°C temperature range. Soybeans were fertilized with Osmocote Plus 15–9–12 (ICL Specialty Fertilizers, Summerville, SC, USA) twice a week after true leaves emerged, and received iron chelate micronutrient (Sprint 330 chelated iron 10%, Florham, Park, NJ, USA) every two weeks. Fertilization and micronutrient supplementation ceased during the experimentation period. Twelve soybean genotypes were planted, with 60 plants per genotype. Not all plants germinated, and our experiments were based on 12 genotypes with a total of 498 plants.

### 2.2 Artificial Diet

FAW and SBL larvae were reared on a wheat‐germ–based artificial diet composed of 1000 mL water, 8 g agar, and 200 g of general lepidopteran diet and prepared per the specifications of the manufacturer and previous work (Frontier Agricultural Services, Calexico, CA, USA) (Portman et al. 2015). A glass container with 1000 mL water was placed on a hot plate, and 8 g of agar was added to it and stirred thoroughly. The water was boiled, and 200 g of artificial diet was added. The mixture was stirred thoroughly to avoid clumps, allowed to cool at room temperature to solidify, and stored in the refrigerator.

### 2.3 Experiments

#### 2.3.1 Pulsating Drought Experiment

Soybean plants at V3 stage (*n* = 30 per genotype) were subjected to drought conditions while control plants were watered regularly. The plants experiencing drought treatment were not watered until they reached a soil moisture content range of 10%–15%, which took about 6 days (Gautam, Shafi, and Kariyat 2024). The volumetric water content of the plants was monitored on alternating days using a soil moisture probe (Bluelab Metpulse pulse meter, Bluelab Corporation Limited (NZ)). After the drought treatment, the plants were watered normally for 3 days until they recovered. Exposing the plants to drought conditions and then allowing them to recover, known as pulsating drought, has been shown to be more similar to the drought periods that plants experience in the field (Grinnan, Carter, and Johnson 2013; Gautam, Shafi, and Kariyat 2024).

##### 2.3.1.1 Plant Growth Traits Measured

Plant height, number of leaves, and chlorophyll content were recorded from each plant prior to and immediately after drought, to gain insight into how pulsating drought impacted the growth of soybeans and to compare phenotypic traits of fast‐wilting and slow‐wilting soybean genotypes. Plant height was measured in centimeters from the plant apex to the stem above the soil level. The number of leaves was determined by counting fully opened, trifoliate clusters of leaves. The chlorophyll content of the leaves was obtained by using SPAD 502 Plus Chlorophyll Meter (Spectrum Technologies Inc., Illinois, USA).

#### 2.3.2 Ninety‐Six‐Hour Damage Exposure Experiment

##### 2.3.2.1 Damage Exposure

FAW and SBL larvae were reared on an artificial diet to the second instar for them to reach a weighable mass (Singh and Kariyat 2020). Second instar FAW and SBL larvae were weighed and then individually contained in mesh organza bags (Amazon, Seattle, WA, USA) that were placed around a trifoliate leaf on a plant and removed after 96 h. For each genotype, 10 plants were exposed to FAW herbivory, and 10 plants were exposed to SBL herbivory. Topmost fully developed new trifoliate leaves were selected for herbivory. The caterpillars were placed on the plants 3 days after pulsating drought treatment concluded, and the plants were allowed to recover.

##### 2.3.2.2 Larval Traits Measured

After 96 h of either FAW or SBL herbivory, a damage assessment was conducted on the leaves that were fed on by FAW. Leaf damage was determined on a scale of 0 to 4, with 0 representing no damage and 4 representing complete defoliation (Chavana et al. 2021). In addition to the damage assessment, the FAW and SBL larvae were weighed prior, and post being placed on the soybean plants and percentage mass gain was calculated using the equation $\frac{\left(\text{Final mass}-\text{Initial mass}\right)}{\text{Initial mass}}\times 100$.

##### 2.3.2.3 Plant Growth Traits Measured Post Herbivory

Seven days pre and post application of FAW and SBL larvae, plant height, number of leaves, and chlorophyll content of each plant were recorded to compare traits of fast‐wilting and slow‐wilting soybean genotypes. To further examine the impact of drought and herbivory on soybean physiology, we used a portable photosynthesis system model LI‐6400/XT (LI‐COR Biosciences, Lincoln, NE, USA) on soybean plants in the V6 stage once they had been exposed to both drought and herbivory. The settings during the experiments included 1500 μmol m^−2^ s^−1^ photosynthetically active photon flux, environment CO_2_ concentration of 400 mol m^−2^ s^−1^, and chamber humidity between 45% and 55% (Gautam, Shafi, and Kariyat 2024). The traits measured were photosynthesis (μmmol CO_2_ m^−2^ s^−1^), stomatal conductance (mol m^−2^ s^−1^), and transpiration (mmol H_2_O m^−2^ s^−1^). The measurements were carried out between 10:00 AM and 1:00 PM on a sunny day with an average temperature of 26°C on 6 plants randomly chosen from each treatment from 6 genotypes for a total of 144 plants.

### 2.4 Yield Data

Once the soybean plants reached full maturity, the pods were harvested to examine the impacts of drought and herbivory on yield. The pods were counted for each treatment. A total of 100 pods were randomly selected from each treatment and weighed. Then the number of seeds in each pod was counted. Ninety seeds were randomly selected from each treatment for each genotype and were weighed. From this, 30 seeds were randomly selected from each treatment for each genotype, and a digital vernier caliper (Zhjan, Qingdao Hezhong Trading Company, Qingdao, Shandong, China) was used to measure the seed diameter.

### 2.5 Germination Rate Post Herbivory and Drought Treatments

Of the harvested seeds, 32 seeds were sown per treatment for each genotype and the number of seeds germinated was counted each morning and evening to determine fitness of the seeds after experiencing drought and herbivory treatments.

### 2.6 Statistical Analysis

Plant traits such as height, number of leaves, and chlorophyll were subtracted from each stage prior to ascertain the changes between stages rather than the total height, number of leaves, and chlorophyll at each stage, allowing a more accurate examination of the impact of each treatment throughout the study. Plant height, number of new leaves, and change in chlorophyll content to timeframe and either wilting speed or treatment type, the data were analyzed using the Wilcoxon/Kruskal–Wallis nonparametric test due to the data not being normal after performing a Shapiro–Wilks normality test. Dunn's comparisons were then used for pairwise comparisons. We used ANOVA to analyze pod traits and larval mass gain with wilting speed and treatment as effects alongside timeframe. Tukey's post hoc test was then used for pairwise comparisons. When comparing net photosynthesis, stomatal conductance, transpiration, yield traits, leaf damage, larval mass gain, or percent germination across fast‐ or slow‐wilting soybean plants, we used *t* test. We used Tukey's post hoc tests when comparing net photosynthesis, stomatal conductance, transpiration, yield traits, leaf damage, larval mass gain, or percent germination for pairwise comparisons across drought and herbivory treatments. When comparing the number of seeds germinated by date, we used a generalized regression with wilting speed and treatment as effects following a Poisson distribution.

## 3 Results

### 3.1 Changes in Plant Growth Traits Across Wilting Speeds

#### 3.1.1 Plant Height

**FIGURE 1:**
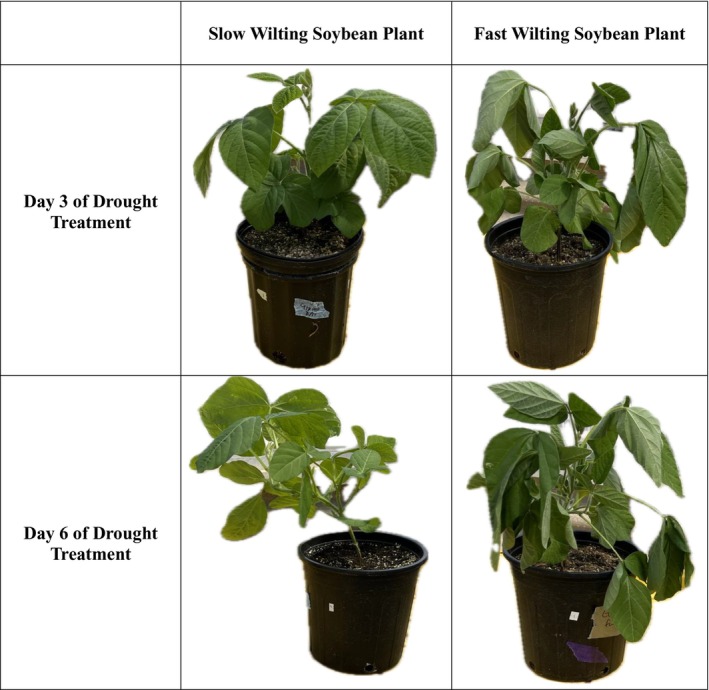
A cross box depicting the visual difference between fast‐ and slow‐wilting soybean genotypes after 3 days and 6 days of drought treatment.

**FIGURE 2:**
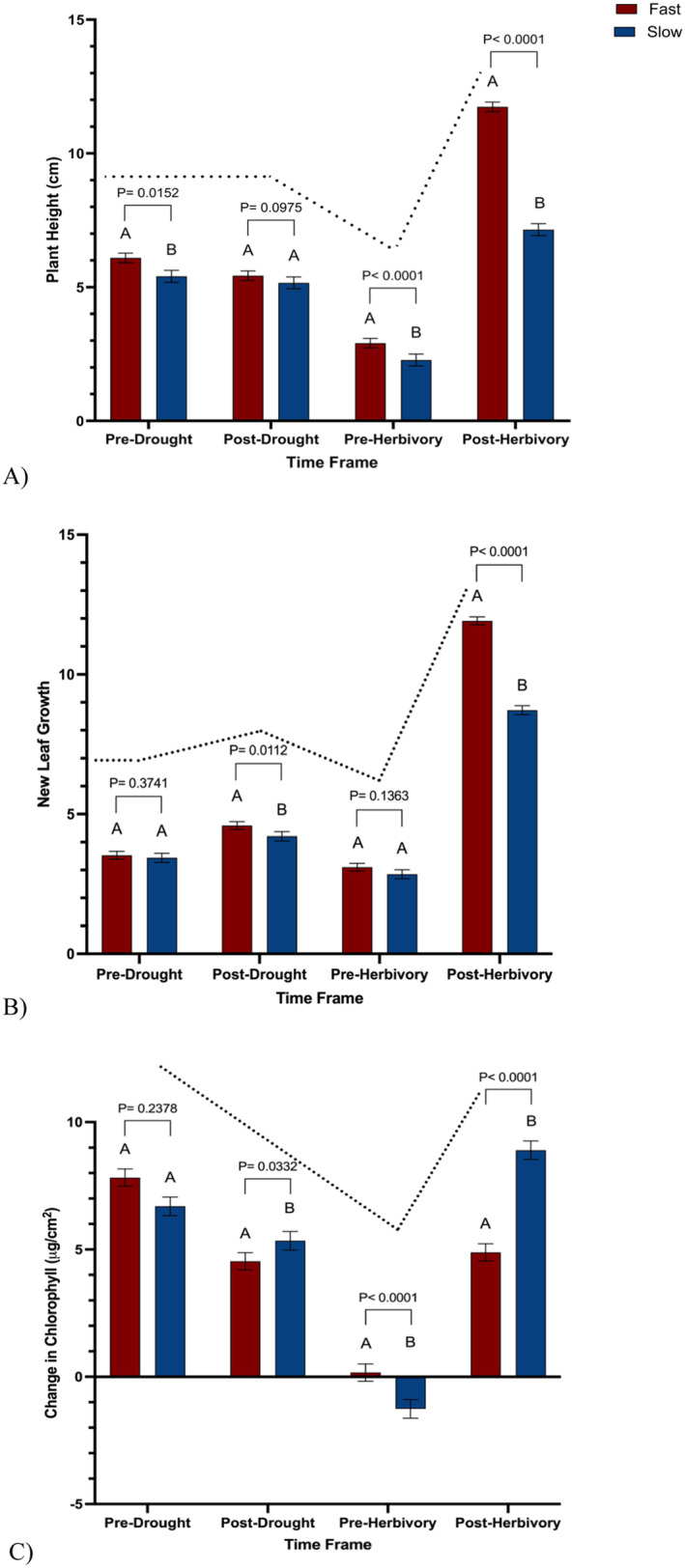
Changes in plant traits over the course of the study. Plants that experienced pulsating drought were also exposed to insect herbivory from either fall armyworm (FAW) or soybean looper (SBL). (A) Change in plant height, (B) difference in number of leaves, and (C) change in mean chlorophyll content over the course of the study across fast‐ and slow‐wilting soybean genotypes (Kruskal–Wallis; *p* < 0.0001). Different letters denote significant differences in mean mass at the 5% level of significance, and data are presented as mean ± SE (standard error). *N* = 498; number of plants per treatment was 30.

There were significant visual differences across fast and slow‐wilting soybean genotypes throughout the study (Figure 1). Overall, fast‐wilting plants were taller ($\bar{X}$  = 6.54 ± 0.12) than slow‐wilting soybean plants ($\bar{X}$ = 4.99 ± 0.14) (Figure 2A, *p* < 0.0001). Prior to any drought treatment, fast‐wilting soybean genotypes grew significantly more in height than ($\bar{X}$ = 6.09 ± 0.18) slower‐wilting ($\bar{X}$ = 5.41 ± 0.22) soybean genotypes (Figure 2A, *p* = 0.015). Immediately post drought treatment, there was no significant difference in height between fast‐wilting ($\bar{X}$ = 5.43 ± 0.18) plants and slow‐wilting ($\bar{X}$  = 5.16 ± 0.22) plants (Figure 2A, *p* = 0.098). Before the herbivores were released (pre herbivory treatment) and once the plants were recovered from drought, fast‐wilting soybean ($\bar{X}$ = 2.91 ± 0.18) plants had significantly more plant height than slow‐wilting soybean ($\bar{X}$ = 2.28 ± 0.22) plants (Figure 2A, *p* < 0.0001). Post herbivory treatment, fast‐wilting soybean ($\bar{X}$ = 11.74 ± 0.18) plants grew significantly taller than slow‐wilting soybean ($\bar{X}$ = 7.15 ± 0.22) plants (Figure 2A, *p* < 0.0001). Fast‐wilting plants were taller than slow‐wilting soybean plants regardless of timeframe.

#### 3.1.2 New Leaf Growth

Fast‐wilting soybean plants ($\bar{X}$ = 5.78 ± 0.12) had more new leaves than slow‐wilting soybean ($\bar{X}$  = 4.80 ± 0.13) plants (Figure 2B, *p* < 0.0001). Pre drought treatment, there was no significant difference in the number of new leaves between fast‐wilting ($\bar{X}$ = 3.53 ± 0.14) and slower‐wilting ($\bar{X}$  = 3.44 ± 0.16) soybean genotypes (Figure 2B, *p* = 0.37). Immediately following drought treatments (post drought), fast‐wilting soybean ($\bar{X}$ = 4.59 ± 0.14) plants grew significantly more new leaves than slow‐wilting soybean ($\bar{X}$ = 4.21 ± 0.16) plants (Figure 2B, *p* = 0.01). Once soybean plants were recovered, and prior to herbivory treatment, there was no significant difference in the number of new leaves between fast‐ ($\bar{X}$ = 3.10 ± 0.14) and slow‐wilting soybean ($\bar{X}$ = 2.85 ± 0.16) plants (Figure 2B, *p* = 0.14). Following herbivory treatment, fast‐wilting soybean ($\bar{X}$ = 11.92 ± 0.14) plants grew significantly more leaves than slow‐wilting soybean ($\bar{X}$ = 8.72 ± 0.16) plants (Figure 2B, *p* < 0.0001). Overall, fast‐wilting plants had more new leaf growth than slow‐wilting soybean plants regardless of the developmental stage of the plant.

#### 3.1.3 Chlorophyll Content

Fast‐wilting soybean plants ($\bar{X}$ = 4.35 ± 0.20) had lower chlorophyll content than slow‐wilting soybean ($\bar{X}$  = 4.92 ± 0.21) plants (Figure 2C, *p* = 0.03). Prior to any drought treatment (pre drought), there was no significant difference in the chlorophyll content between fast‐wilting soybean plants ($\bar{X}$ = 7.83 ± 0.33) and slower‐wilting ($\bar{X}$ = 6.69 ± 0.37) soybean genotypes (Figure 2B, *p* = 0.24). Immediately post drought treatments, fast‐wilting soybean ($\bar{X}$ = 4.54 ± 0.33) plants had a significantly lower increase in chlorophyll content than slow‐wilting soybean ($\bar{X}$ = 5.34 ± 0.37) plants (Figure 2C, *p* = 0.03). Following drought recovery and prior to herbivory treatments, fast‐wilting soybean ($\bar{X}$ = 0.17 ± 0.34) plants had a significant increase in chlorophyll content than slow‐wilting soybean ($\bar{X}$ = −1.26 ± 0.37) plants which experienced a decrease (Figure 2C, *p* < 0.0001). Post herbivory treatments, fast‐wilting soybean ($\bar{X}$ = 4.89 ± 0.34) plants have significantly less chlorophyll content than slow‐wilting soybean ($\bar{X}$ = 8.89 ± 0.37) plants (Figure 2C, *p* < 0.0001). Overall, there was a significant difference in chlorophyll content between fast‐ and slow‐wilting soybean genotypes.

### 3.2 Changes in Plant Growth Traits Across Drought and Herbivory Treatments

#### 3.2.1 Plant Height

**FIGURE 3:**
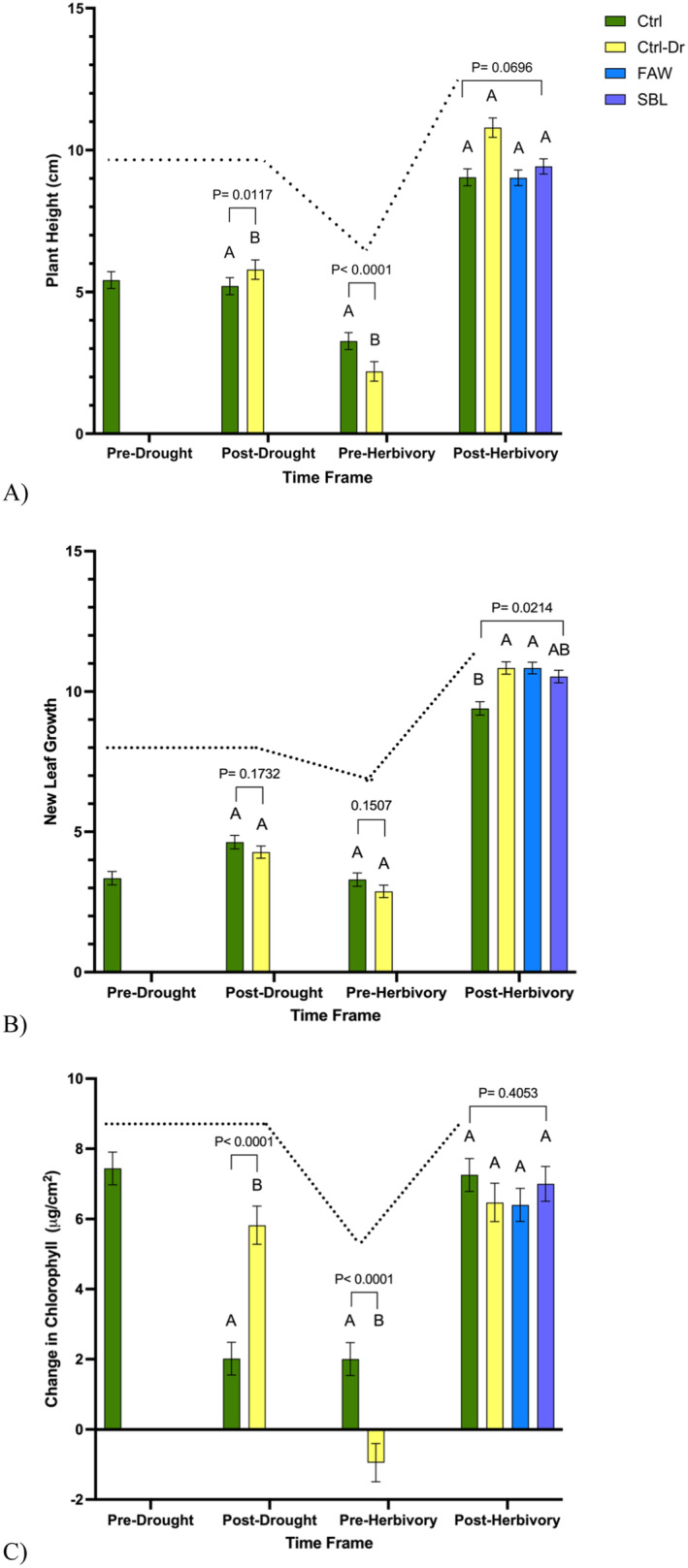
Changes in plant traits over the course of the study. “Ctrl” represents control plants, “Ctrl‐Dr” represent plants that only received drought treatment, “FAW” represents plants that received drought treatment and fall armyworm herbivory, and “SBL” represent plants that received drought treatment and soybean looper herbivory. Note that throughout the timeframe, not all treatments are represented until the specified variable is altered. (A) Change in mean plant height growth (*p* = 0.02), (B) change in mean number of leaves (*p* = 0.98), and (C) change in mean chlorophyll content (*p* = 0.88) over the course of the study across drought and herbivory treatments (Kruskal–Wallis test). Different letters denote significant differences in mean mass at the 5% level of significance, and data are presented as mean ± SE (standard error). *N* = 498, and *N* per treatment was 15–30.

Immediately post drought treatment, plants that only experienced drought ($\bar{X}$ = 5.2 ± 0.30) had significantly more plant height than plants that did not experience drought or herbivory treatment (control) ($\bar{X}$  = 5.78 ± 0.34) (Figure 3A, *p* = 0.01). Before herbivory treatment (pre herbivory) and after plants recovered from drought, plants that did not experience drought or herbivory treatments ($\bar{X}$ = 3.27 ± 0.30) grew significantly more than plants that only experienced drought (control drought) ($\bar{X}$  = 2.20 ± 0.34) (Figure 3A, *p* < 0.0001). After herbivory treatment (post herbivory), there was no significant difference in plant height between plants that did not experience drought or herbivory treatment ($\bar{X}$ = 9.04 ± 0.30), plants that only experienced drought treatments ($\bar{X}$ = 10.79 ± 0.34), plants that experienced drought treatment and FAW herbivory ($\bar{X}$ = 9.02 ± 0.28), and plants that experienced drought and SBL herbivory treatment ($\bar{X}$ = 9.42 ± 0.27) (Figure 3A, *p* = 0.07). Overall, there was a significant difference in plant height between treatments over the course of the study (Figure 3A, *p* = 0.02).

#### 3.2.2 New Leaf Growth

There was no significant difference in new leaf growth between control plants ($\bar{X}$ = 4.63 ± 0.24) and plants that only experienced drought ($\bar{X}$ = 4.28 ± 0.21) immediately following drought treatment (Figure 3B, *p* = 0.17). Once soybean plants were recovered and prior to herbivory treatment, there was no significant difference in new leaf growth between control plants ($\bar{X}$ = 4.30 ± 0.24) and plants that only experienced drought ($\bar{X}$ = 2.88 ± 0.22) (Figure 3B, *p* = 0.15). Post herbivory treatment, plants that only experienced drought treatment ($\bar{X}$  = 10.84 ± 0.21) and plants that experienced drought treatment and FAW herbivory ($\bar{X}$ = 10.84 ± 0.21) had the most growth while plants that did not experience drought or herbivory treatments ($\bar{X}$  = 9.39 ± 0.23) experienced the least new leaf growth (Figure 3B, *p* = 0.02). No significant difference was observed between treatments over the course of the study (Figure 3A, *p* = 0.98).

#### 3.2.3 Chlorophyll Content

Immediately after drought treatment, plants that only experienced drought treatment ($\bar{X}$ = 2.02 ± 0.47) had a significantly higher increase in chlorophyll content than plants that did not experience drought treatment ($\bar{X}$ = 5.82 ± 0.55) (Figure 3C, *p* < 0.0001). Soybean plants that only experienced drought treatment ($\bar{X}$ = −0.95 ± 0.55) had a significant decrease in chlorophyll content compared to control plants ($\bar{X}$ = 2.00 ± 0.47) once recovered and prior to herbivory treatment (Figure 3C, *p* < 0.0001). Following herbivory treatments, there was no significant difference in the chlorophyll content among control plants ($\bar{X}$ = 7.25 ± 0.47), plants that only experienced drought ($\bar{X}$ = 6.47 ± 0.55), plants that experienced drought and FAW herbivory ($\bar{X}$ = 6.40 ± 0.47), and plants that experienced drought and SBL herbivory ($\bar{X}$  = 7.00 ± 0.50) (Figure 3C, *p* = 0.41). Overall, there was no significant difference in chlorophyll content between treatments over the course of the study (Figure 3A, *p* = 0.88).

### 3.3 Impact of Wilting Speed and Drought and Herbivory Treatment on Larval Traits

**FIGURE 4:**
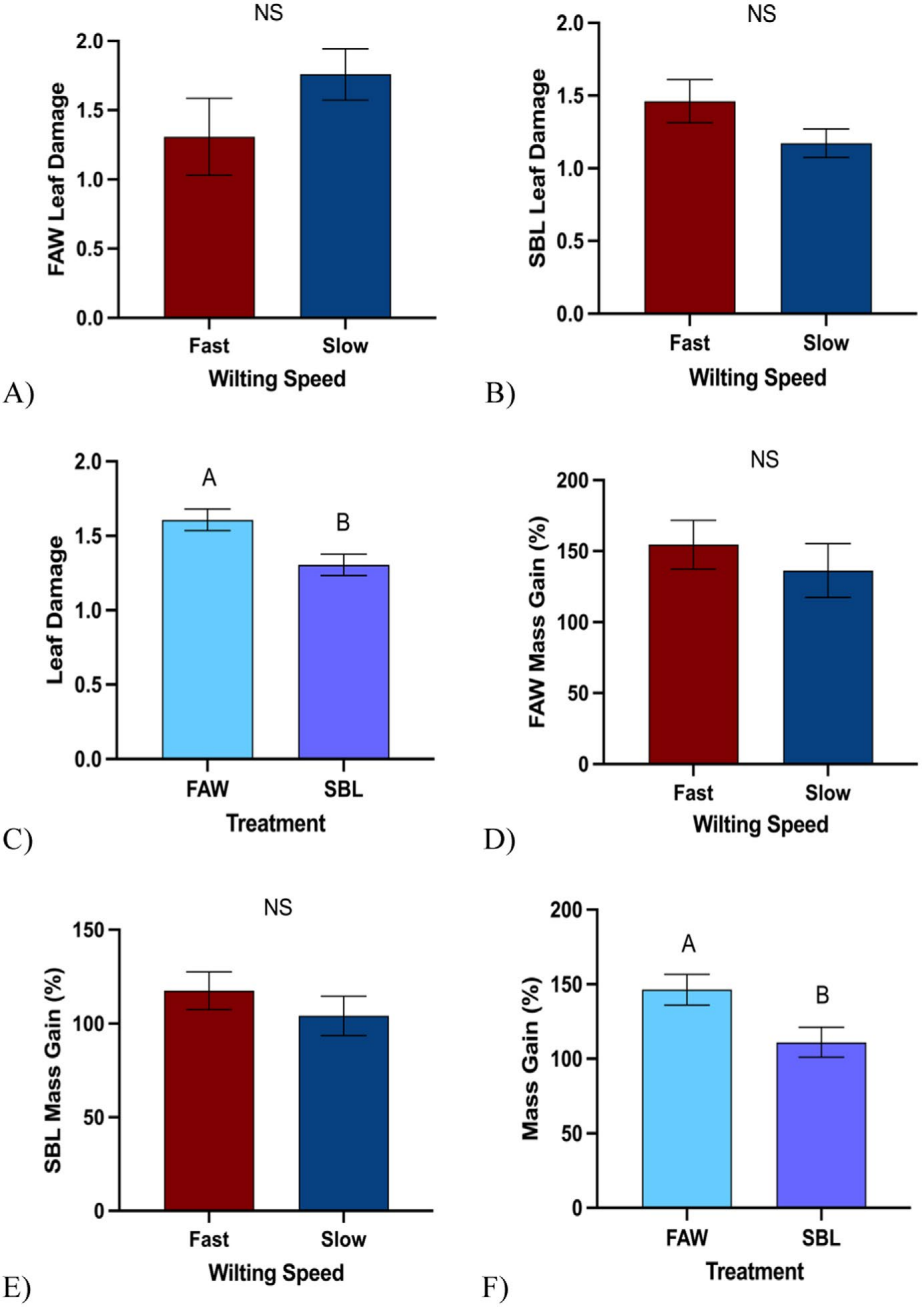
Herbivory traits. (A) Mean leaf damage inflicted by fall armyworm (FAW) (*p* = 0.12), (B) mean leaf damage inflicted by soybean looper (SBL) (*p* = 0.24), (C) mean leaf damage inflicted by herbivores on droughttreated plants (*p* = 0.003), (D) mean percent mass gained by FAW (*p* = 0.47), (E) mean percent mass gained by SBL (*p* = 0.36), and (F) Mean mass gained by both herbivores on drought‐treated plants (*p* = 0.02) across both fast‐ and slow‐wilting soybean genotypes (*t* test). NS represents nonsignificant results while different letters denote significant differences in mean mass at the 5% level of significance, and data are presented as mean ± SE (standard error). *N* = 247. For each treatment per herbivore species, *N* = 10.

**FIGURE 5:**
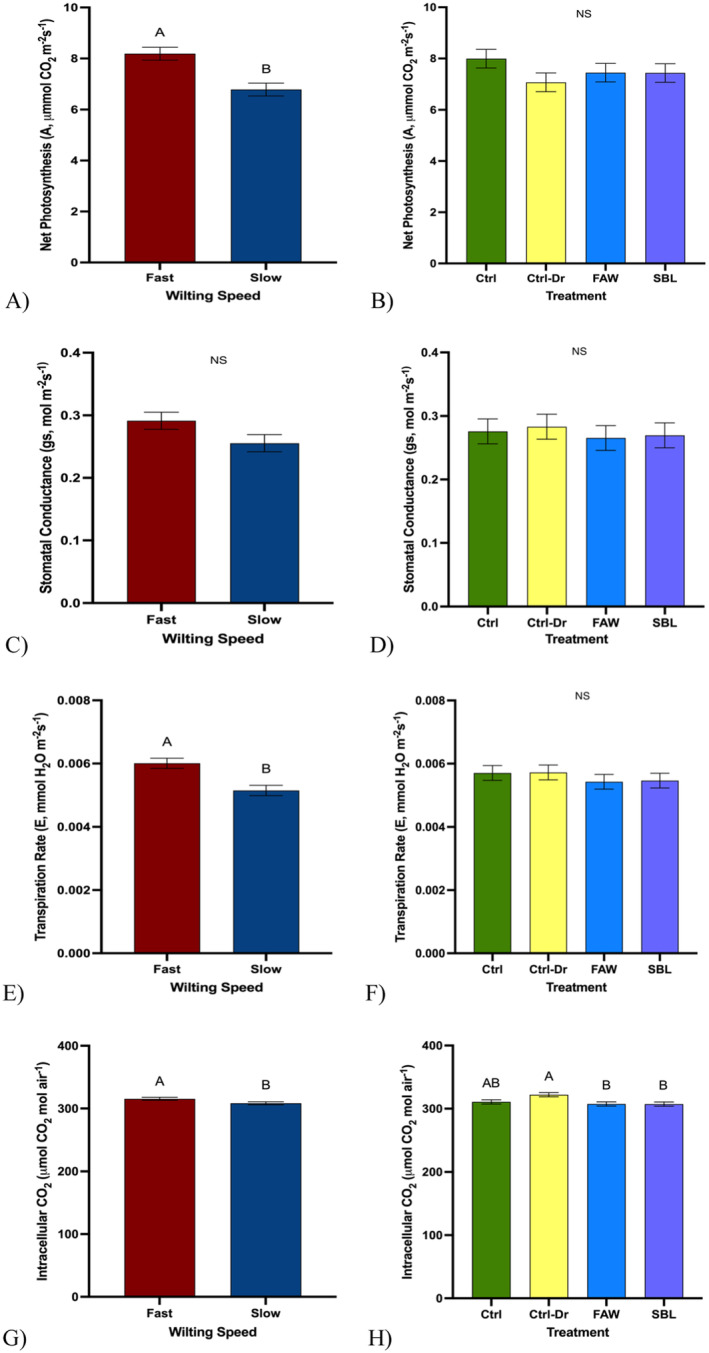
Soybean physiology traits. “Ctrl” represents control plants, “Ctrl‐Dr” represents plants that only received drought treatment, “FAW” represents plants that received drought treatment and fall armyworm herbivory, and “SBL” represents plants that received drought treatment and soybean looper herbivory. (A) Mean net photosynthesis across fast‐ and slow‐wilting soybean genotypes (*t* test; *p* < 0.0001). (B) Mean net photosynthesis across drought and herbivory treatments [fall armyworm (FAW) or soybean looper (SBL)] (ANOVA; *p* = 0.35. (C) Mean stomatal conductance across fast‐ and slow‐wilting soybean genotypes (*t* test; *p* = 0.07). (D) Mean stomatal conductance across drought and herbivory treatments (ANOVA; *p* = 0.93). (E) Mean transpiration rate across fast‐ and slow‐wilting soybean genotypes (*t* test; *p* = 0.0002). (F) Mean transpiration rate across drought and herbivory treatments (ANOVA; *p* = 0.73). (G) Mean intracellular CO_2_ across fast‐ and slow‐wilting soybean genotypes (*t* test; *p* = 0.04). (H) Mean intracellular CO_2_ across drought and herbivory treatments (ANOVA; *p* = 0.005). Different letters denote significant differences in mean mass at the 5% level of significance, and data are presented as mean ± SE (standard error). *N* = 144. For each treatment per genotype (three fast and three slow), six plants were randomly sampled.

FAW larvae ($\bar{X}$ = 1.61 ± 0.07) inflicted more damage than SBL larvae ($\bar{X}$ = 1.30 ± 0.07) (Figure 4A, *p* = 0.003). However, there were no significant differences in the damage inflicted by FAW larvae between fast‐ ($\bar{X}$ = 1.48 ± 0.12) and slow‐ ($\bar{X}$ = 1.76 ± 0.13) wilting soybean genotypes (Figure 4B, *p* = 0.12). Similarly, there were no significant differences in the damage inflicted by SBL larvae between fast‐ ($\bar{X}$ = 1.36 ± 0.07) and slow‐ ($\bar{X}$ = 1.24 ± 0.08) wilting soybean genotypes (Figure 4C, *p* = 0.24). FAW larvae ($\bar{X}$ = 146.35 ± 10.30) gained more mass overall than SBL larvae ($\bar{X}$ = 111.17 ± 10.13) (Figure 4D, *p* = 0.02). However, there were no significant differences in the percent mass gain of FAW larvae between fast‐ ($\bar{X}$ = 154.58 ± 17.09) and slow‐ ($\bar{X}$ = 136.28 ± 18.90) wilting soybean genotypes (Figure 5E, *p* = 0.47). And there were no significant differences in the percent mass gain by SBL larvae between fast‐ ($\bar{X}$ = 117.54 ± 9.97) and slow‐ ($\bar{X}$ = 104.14 ± 10.47) wilting soybean genotypes (Figure 5F, *p* = 0.36). FAW ate more leaf tissue than SBL regardless of wilting speed and gained more mass than SBL.

### 3.4 Soybean Physiology Traits

Fast‐wilting soybean plants had a higher net photosynthesis ($\bar{X}$ = 8.19 ± 0.25) than slow‐wilting soybean plants ($\bar{X}$ = 6.79 ± 0.25) (Figure 5A, *p* < 0.0001). There was no significant difference in net photosynthesis in soybean control plants ($\bar{X}$ = 7.99 ± 0.36), plants that only experienced drought treatment ($\bar{X}$ = 7.07 ± 0.37), plants that experienced drought and FAW herbivory treatment ($\bar{X}$ = 7.45 ± 0.36), and plants that experienced drought and SBL herbivory treatment ($\bar{X}$ = 7.43 ± 0.36) (Figure 5B, *p* = 0.35). No significant difference in stomatal conductance between fast‐wilting soybean plants ($\bar{X}$ = 0.29 ± 0.01) and slow‐wilting soybean plants ($\bar{X}$ = 0.26 ± 0.01) was observed (Figure 5C, *p* = 0.07). There was no significant difference in stomatal conductance among control plants ($\bar{X}$ = 0.28 ± 0.02), plants that only experienced drought treatment ($\bar{X}$ = 0.28 ± 0.02), plants that experienced drought and FAW herbivory treatment ($\bar{X}$ = 0.27 ± 0.02), and plants that experienced drought and SBL herbivory treatment ($\bar{X}$ = 0.27 ± 0.02) (Figure 5D, *p* = 0.93). Fast‐wilting soybean plants had a higher transpiration rate ($\bar{X}$ = 0.01 ± 0.0002) than slow‐wilting soybean plants ($\bar{X}$ = 0.01 ± 0.0002) (Figure 5E, *p* = 0.0002). There was no significant difference in the transpiration rate among control plants ($\bar{X}$ = 0.01 ± 0.0002), plants that only experienced drought treatment ($\bar{X}$ = 0.01 ± 0.0002), plants that experienced drought and FAW herbivory treatment ($\bar{X}$ = 0.01 ± 0.0002), and plants that experienced drought and SBL herbivory treatment ($\bar{X}$ = 0.01 ± 0.0002) (Figure 5F, *p* = 0.73). Fast‐wilting soybean plants had higher intracellular CO_2_ ($\bar{X}$ = 315.41 ± 2.38) than slow‐wilting soybean plants ($\bar{X}$ = 308.37 ± 2.38) (Figure 5G, *p* = 0.04). Soybean plants that only experienced drought treatment had the highest amount of intracellular CO_2_ ($\bar{X}$ = 322.16 ± 3.35), control plants ($\bar{X}$ = 310.78 ± 3.32) had the second highest amount, and then plants that experienced drought and FAW herbivory treatment ($\bar{X}$ = 307.51 ± 3.30) or drought and SBL herbivory treatment ($\bar{X}$ = 307.31 ± 3.32) had the least amount of intracellular CO_2_ (Figure 5H, *p* = 0.005). Fast‐wilting soybean plants had a higher net photosynthesis and transpiration rate than slow‐wilting soybean plants.

### 3.5 Changes in Fitness Traits in Wilting Speeds Across Drought and Herbivory Treatments

#### 3.5.1 Soybean Pod Yield

**FIGURE 6:**
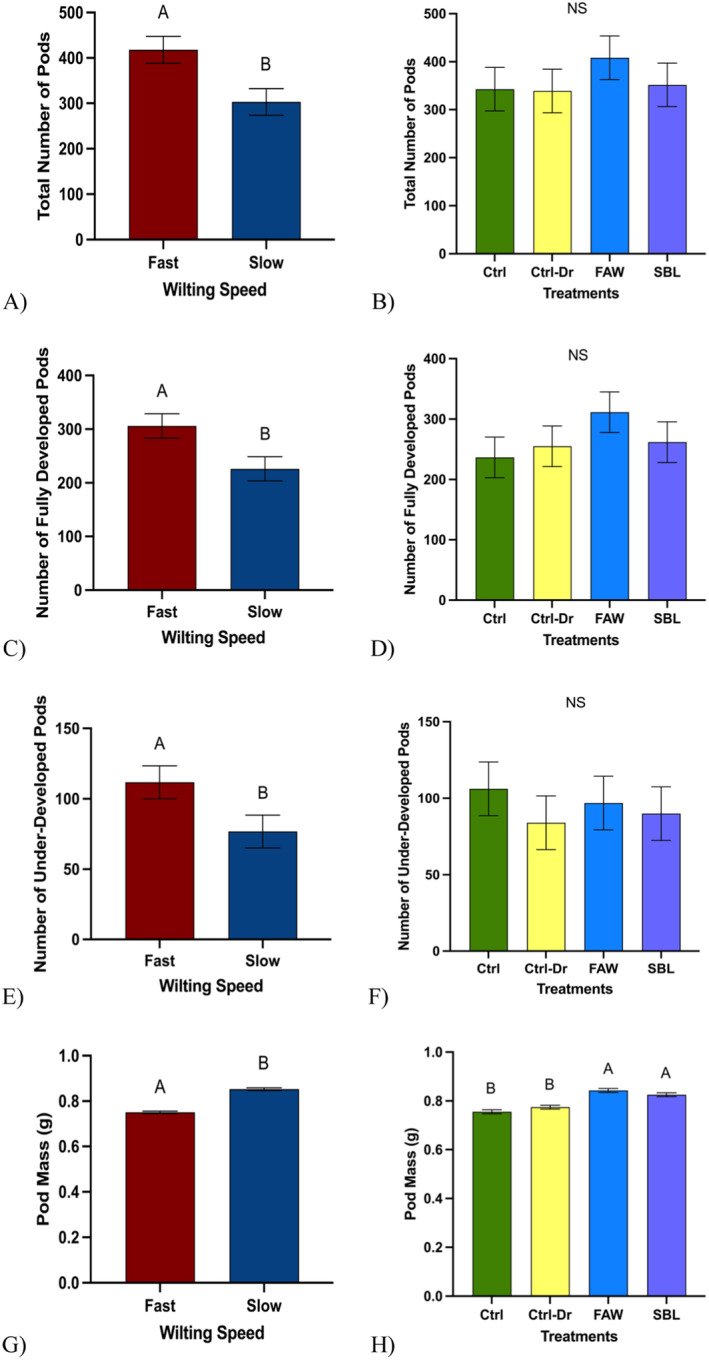
Yield traits: Pods. “Ctrl” represents control plants, “Ctrl‐Dr” represents plants that only received drought treatment, “FAW” represents plants that received drought treatment and fall armyworm herbivory, and “SBL” represents plants that received drought treatment and soybean looper herbivory. (A) Mean number of total pods across fast‐ and slow‐wilting soybean genotypes (*t* test; *p* = 0.01). (B) Mean number of total pods across drought and herbivory treatments (ANOVA; *p* = 0.68). (C) Mean number of fully developed soybean pods across fast‐ and slow‐wilting soybean genotypes (*t* test; *p* = 0.02). (D) Mean number of fully developed soybean pods across drought and herbivory treatments (ANOVA; *p* = 0.45). (E) Mean number of underdeveloped soybean pods across fast‐ and slow‐wilting soybean genotypes (*t* test; *p* = 0.04). (F) Mean number of underdeveloped soybean pods across drought and herbivory treatments (Student ANOVA; *p* = 0.83). (G) Mean soybean pod mass across fast‐ and slow‐wilting soybean genotypes (*t* test; *p* < 0.0001). (H) Mean soybean pod mass across drought and herbivory treatments (ANOVA; *p* < 0.0001. Different letters denote significant differences in mean mass at the 5% level of significance, and data are presented as mean ± SE (standard error). For pod numbers, we collected data from all the plants, and for pod mass, we collected data from 4800 pods, from 100 pods randomly selected from each treatment.

Fast‐wilting soybean plants had significantly more pods in total ($\bar{X}$ = 417.96 ± 29.63) than slow‐wilting soybean plants ($\bar{X}$ = 303.08 ± 29.63) (Figure 6A, *p* = 0.09). There was no significant difference in the total number of pods among control plants ($\bar{X}$ = 342.83 ± 45.44), plants that experienced only drought treatments ($\bar{X}$ = 339.08 ± 45.44), plants that experienced drought and FAW herbivory ($\bar{X}$ = 408.33 ± 45.44), and plants that experienced drought and SBL herbivory treatment ($\bar{X}$ = 351.83 ± 45.44) (Figure 6B, *p* = 0.68). Fast‐wilting soybean plants had significantly more fully developed pods ($\bar{X}$ = 306.17 ± 22.52) compared to slow‐wilting soybean plants ($\bar{X}$ = 226.33 ± 22.52) (Figure 6C, *p* = 0.02). There was no significant difference among control plants ($\bar{X}$ = 236.67 ± 33.70), plants that only experienced drought treatment ($\bar{X}$ = 255.08 ± 33.70), plants that experienced drought and FAW herbivory treatment ($\bar{X}$ = 311.42 ± 33.70), and plants that experienced drought and SBL herbivory treatment ($\bar{X}$ = 261.83 ± 33.70) (Figure 6D, *p* = 0.45). Fast‐wilting soybean plants had significantly more underdeveloped pods ($\bar{X}$ = 111.79 ± 11.67) than slow‐wilting soybean plants ($\bar{X}$ = 76.75 ± 11.67) (Figure 6E, *p* = 0.04). No significant differences were observed among control plants ($\bar{X}$ = 106.17 ± 17.51), plants that only experienced drought treatment ($\bar{X}$= 84 ± 17.51), plants that experienced drought and FAW herbivory treatment ($\bar{X}$ = 96.92 ± 17.51), and plants that experienced drought and SBL herbivory treatment ($\bar{X}$ = 90 ± 17.51) (Figure 6F, *p* = 0.83). Pods from slow‐wilting soybean plants weighed significantly more ($\bar{X}$ = 0.85 ± 0.01) than pods from fast‐wilting plants ($\bar{X}$ = 0.75 ± 0.01) (Figure 6G, *p* < 0.0001). Pods from soybean plants that experienced drought and FAW treatment ($\bar{X}$ = 0.84 ± 0.01) and drought and SBL herbivory treatment ($\bar{X}$ = 0.83 ± 0.01) weighed significantly more than pods from control plants ($\bar{X}$ = 0.76 ± 0.01) or only experienced drought treatment ($\bar{X}$ = 0.77 ± 0.01) (Figure 6H, *p* < 0.0001). Overall, fast‐wilting plants produced more pods than slow‐wilting plants, but pods from slow‐wilting pods had a higher mass.

#### 3.5.2 Soybean Seed Yield

**FIGURE 7:**
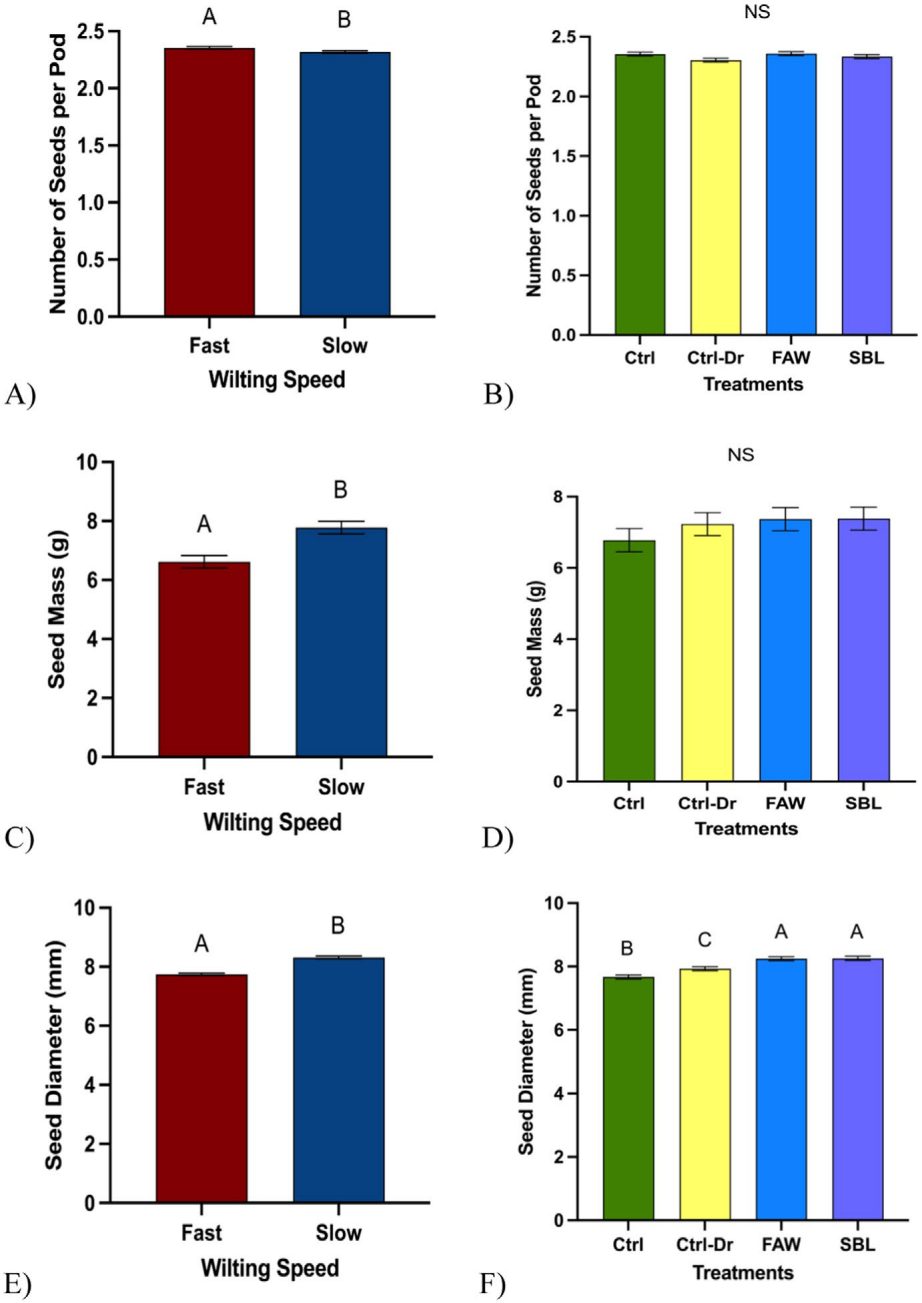
Yield traits: Seeds. “Ctrl” represents control plants, “Ctrl‐Dr” represents plants that only received drought treatment, “FAW” represents plants that received drought treatment and fall armyworm herbivory, and “SBL” represents plants that received drought treatment and soybean looper herbivory. (A) Mean number of seeds per pod across fast‐ and slow‐wilting soybean genotypes (*t* test; *p* = 0.03). (B) Mean number of seeds per pod across drought and herbivory treatments (ANOVA; *p* = 0.08). (C) Mean seed mass across fast‐ and slow‐wilting soybean genotypes (*t* test; *p* = 0.0002). (D) Mean seed mass across drought and herbivory treatments (ANOVA; *p* = 0.52). (E) Mean seed diameter across fast‐ and slow‐witling soybean genotypes (*t* test; *p* < 0.0001). (F) Mean seed diameter across drought and herbivory treatments (ANOVA; *p* < 0.0001). Different letters denote significant differences in mean mass at the 5% level of significance, and data are presented as mean ± SE (standard error). The pods were collected from all 12 genotypes (*N* = 498), whereas 30 seeds were used to measure the diameter from each treatment from 12 genotypes (*N* = 1440).

Pods from fast‐wilting soybean plants had significantly more seeds per pod ($\bar{X}$ = 2.36 ± 0.01) than pods from slow‐wilting soybean plants ($\bar{X}$ = 2.32 ± 0.01) (Figure 7A, *p* = 0.03). No significant difference was observed in the number of seeds per pod in control plants ($\bar{X}$ = 2.35 ± 0.02), plants that only experienced drought treatment ($\bar{X\;}$= 2.30 ± 0.16), plants that experienced drought and FAW herbivory treatment ($\bar{X}$ = 2.36 ± 0.02), and plants that experienced drought and SBL herbivory treatment ($\bar{X}$ = 2.33 ± 0.02) (Figure 7B, *p* = 0.08). Slow‐wilting soybean seeds had a higher mass ($\bar{X}$ = 7.78 ± 0.22) than fast‐wilting soybean seeds ($\bar{X}$ = 6.62 ± 0.21) (Figure 7C, *p* = 0.0002). There was no significant difference in the seed mass of control plants ($\bar{X}$ = 6.78 ± 0.33), plants that only experienced drought treatment ($\bar{X}$ = 7.23 ± 0.32), plants that experienced drought and FAW herbivory treatment ($\bar{X}$ = 7.37 ± 0.32), and plants that experienced drought and SBL herbivory treatment ($\bar{X}$ = 7.39 ± 0.32) (Figure 7D, *p* = 0.52). Seeds from slow‐wilting soybean plants had a significantly larger diameter ($\bar{X}$ = 8.32 ± 0.04) than seeds from fast‐wilting soybean plants ($\bar{X}$ = 7.75 ± 0.04) (Figure 7E, *p* < 0.0001). Seeds from soybean plants that experienced drought and FAW herbivory treatment ($\bar{X}$ = 8.25 ± 0.06) and drought and SBL herbivory treatment ($\bar{X}$ = 8.26 ± 0.06) had a larger diameter than seeds from soybean plants that only experienced drought treatment ($\bar{X}$ = 7.93 ± 0.06) and seeds from control plants had the smallest diameter ($\bar{X}$ = 7.67 ± 0.06) (Figure 7F, *p* < 0.0001). Fast‐wilting soybean plants had more seeds per pod, but slow‐wilting plants had larger seeds with a higher mass.

### 3.6 Germination Experiment

**FIGURE 8:**
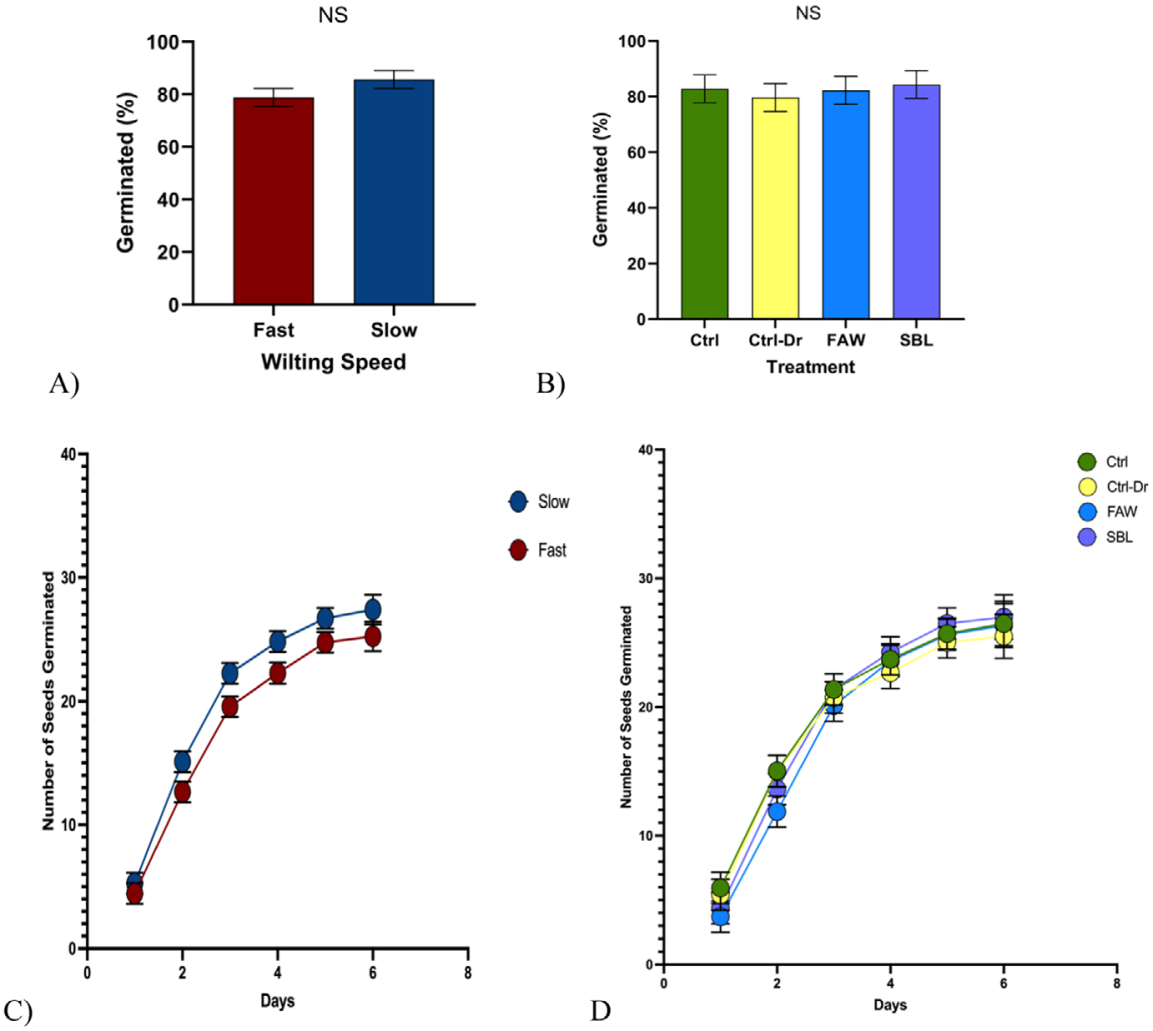
Germination study. “Ctrl” represents control plants, “Ctrl‐Dr” represents plants that only received drought treatment, “FAW” represents plants that received drought treatment and fall armyworm herbivory, and “SBL” represents plants that received drought treatment and soybean looper herbivory. (A) Mean percent germination of seeds from fast‐wilting plants and slow‐wilting plants (*t* test; *p* = 0.17). (B) Mean percent germination of seeds from plants across drought and herbivory treatments (ANOVA; *p* = 0.93). (C) Total number of seeds germinated from fast‐wilting plants and slow‐wilting plants over 6 days (generalized regression with Poisson distribution; *p* = 0.89). (D) Total number of seeds germinated from plants across drought and herbivory treatments over 6 days (generalized regression with Poisson distribution; *p* = 0.98). Different letters denote significant differences in mean mass at the 5% level of significance, and data are presented as mean ± SE (standard error). 32 seeds were randomly selected per treatment per genotype for the germination assay to a total of 1264 total seeds planted.

Fast‐wilting ($\bar{X}$ = 78.91 ± 3.42) and slow‐wilting soybean plants ($\bar{X}$ = 85.68 ± 3.42) (Figure 8A, *p* = 0.17) had no significant difference in the germination percentage. No significant differences were observed in the percentage of germination among control plants ($\bar{X}$ = 82.81 ± 5.02), plants that only experienced drought treatment ($\bar{X}$ = 79.69 ± 5.02), plants that experienced drought and FAW herbivory treatment ($\bar{X}$ = 82.29 ± 5.02), and plants that experienced drought and SBL herbivory treatment ($\bar{X}$ = 84.38 ± 5.02) (Figure 8B, *p* = 0.93). There was no significant difference in the number of seeds that germinated each date between seeds from fast‐ and slow‐wilting plants (Figure 8C, *p* = 0.89). No significant difference in the number of seeds that germinated each date between treatments was observed (Figure 8D, *p* = 0.71). There was no significant difference in germination across wilting speed or treatment.

### 3.7 Genotypic Variation in Soybean Growth Traits, Physiological Traits, and Yield Traits

There was a significant difference in plant height, number of new leaves, and chlorophyll content across all genotypes (Figure S1a, *p* < 0.0001; Figure S1b, *p* < 0.0001; and Figure S1c, *p* < 0.0001). There was a significant difference in net photosynthesis, stomatal conductance, transpiration rate, and intracellular CO_2_ between all genotypes examined (Figure S2a, *p* < 0.0001; Figure S2b, *p* < 0.0001; Figure S2c, *p* < 0.0001; and Figure S2d, *p* < 0.0001).

FAW leaf damage was not significantly different across all genotypes (Figure S3a, *p* = 0.64). There was a significant difference in SBL leaf damage across all soybean genotypes (Figure S3b, *p* = 0.04). No significant difference was observed in the percent mass gained by FAW across all genotypes (Figure S3c, *p* = 0.88). Percent mass gained by SBL across all genotypes was not significantly different (Figure S3d, *p* = 0.16). Total number of pods was significantly different across all genotypes (Figure S4a, *p* < 0.0001). There was a significant difference in the number of fully developed pods across all genotypes (Figure S4b, *p* < 0.0001). The number of underdeveloped pods was significantly different across all genotypes (Figure S4c, *p* = 0.0002). Pod mass was significantly different across all genotypes (Figure S4d, *p* < 0.0001). A significant difference was observed in the number of seeds per pod across all genotypes (Figure S5a, *p* < 0.0001). Mean seed mass was significantly different across all genotypes (Figure S5b, *p* < 0.0001). Mean seed diameter was significantly different across all genotypes (Figure S5c, *p* < 0.0001). There was also a significant difference in the percent of seeds germinated across all genotypes (Figure S6a, *p* = 0.0004). A significant difference in the number of seeds that germinated each date across all genotypes was also observed (Figure S6b, *p* = 0.001). Collectively, as expected, we found strong genotypic variation for most traits we measured.

## 4 Discussion

This study examined the interactions of WUE traits under abiotic and biotic stressors, and how it affects herbivory and soybean growth. Understanding the relationship between insect herbivory and WUE traits is vital to maximize soybean yield experiencing both abiotic and biotic stressors in their environment, especially in areas more prone to drought stress where food insecurity is persistent (Khojely et al. 2018). We show that fast‐wilting genotypes produced more pods, but slow‐wilting soybeans produced heavier pods and seeds, indicating that within fitness traits, there are differential effects, as seeds are known to produce more seed oil (Goettel et al. 2022). In terms of herbivore traits, impacts were seen across their life cycle rather than in the immediate aftermath of feeding (Ayala et al. 2024).

In this study, soybean morphological traits, plant height, and number of leaves were higher in fast‐wilting genotypes than in slow‐wilting genotypes. This coincides with previous studies and is an expected trait in fast‐wilting genotypes (Chamarthi et al. 2023). However, immediately after drought and during the recovery period prior to herbivory, both fast‐ and slow‐wilting soybean plants experienced a steep decline in apical growth and leaf production, even losing leaves. Following herbivory treatment, their height and leaf production significantly increased. Seven days after herbivory, slow‐wilting soybean genotypes not only displayed a significantly higher chlorophyll content compared to fast‐wilting soybean genotypes but it also surpassed the amount prior to experiencing any environmental stress. After undergoing drought and herbivory, slow‐wilting soybean genotypes had a higher chlorophyll content than soybean genotypes that had not experienced drought or herbivory. This suggests that when soybeans experience both drought and herbivory concurrently, they overcompensate, consistent with a recent study (Gautam, Shafi, and Kariyat 2024).

Fast‐wilting soybean plants had an overall higher net photosynthesis and intracellular CO_2_ compared to slow‐wilting soybean plants which may explain why fast‐wilting genotypes tend to be taller and have more leaves than slower‐wilting soybean genotypes (Chamarthi et al. 2023). Fast‐wilting soybean plants also exhibited higher rates of transpiration compared to slower‐wilting soybean genotypes. Slow‐wilting genotypes exhibiting lower transpiration rate and net photosynthesis indicate that they are significantly better at optimizing their WUE by lowering these traits (Kunert and Vorster 2020). When we examined the intracellular CO_2_, we saw that plants that experienced both drought and insect herbivory had the lowest intracellular CO_2_ compared to plants that did not experience herbivory. This is primarily attributed to the stress responses of the plants triggering the regulation of stomatal opening which is directly related to the chlorophyll content and intracellular CO_2_ present in the leaves (Anjum et al. 2011). However, periodic measurement of physiological traits in soybeans under drought and herbivory through future experiments is warranted to explore the temporal adjustments these plants undergo under such stressed conditions.

Immediately following drought stress, the plants tended to overcompensate and surpassed the plants that did not experience drought stress. The compensation following the rehydration of soybean plants has been documented in plant growth traits such as height, leaf surface area, root length, and chlorophyll (Dong et al. 2019; Petcu, Bărbieru, and Vlad 2024). Once the plants recovered, there was a significant decline in plant growth and chlorophyll content of the plants that experienced drought stress. However, following herbivory treatment, the plants were able to fully recover and had no significant differences between plant growth and chlorophyll content across all treatments following herbivory. This compensation effect has been documented across plants and occurs when plants exhibit abrupt growth once rehydrated following a period of drought depending on the growth stage of the plants (Dong et al. 2019; Gautam, Shafi, and Kariyat 2024). In leaf production, there was no significant difference in the number of new leaves produced until after herbivory treatment. With plants that were exposed to either only drought treatment or a combination of drought treatment and FAW herbivory had the most leaf growth compared to plants that did not experience drought or herbivory.

While not statistically significant, FAW tended to feed more on slower‐wilting genotypes compared to fast‐wilting genotypes and SBL tended to feed more on faster‐wilting genotypes, but there was no significant difference in mass gain between wilting speeds. In past studies, FAW gained more mass in fast‐wilting soybean genotype, but that was not observed during the course of this study (Grinnan, Carter, and Johnson 2013; Ayala et al. 2024). We speculate that this disparity is due to the drought treatment that the plants experienced as FAW have been recorded to gain less mass on stressed plants compared to unstressed plants (Mubayiwa et al. 2023). Fast‐wilting soybean plants are less able to adapt to drought stress and become less palatable to FAW, causing them to potentially consume less leaf material compared to slow‐wilting soybeans and gain less mass compared to when consuming slow‐wilting soybean plants. In drought conditions, slow‐wilting soybean genotypes exhibit higher levels of nitrogen fixation and nitrogen assimilation, meaning that under drought conditions, fast‐wilting soybean plants may contain fewer nutrients in their leaf tissue (Bellaloui et al. 2013).

Overall, fast‐wilting soybean plants produced more pods than slow‐wilting soybean plants, but the pods produced by slow‐wilting soybean plants tend to have a higher mass. Soybean plants that experienced a combination of drought and herbivory did not have significantly more pods than control soybean plants or soybean plants that only experienced drought. However, the pods produced by plants that faced drought, and herbivory had significantly higher mass than those that only experienced drought. Fast‐wilting soybean pods had significantly more seeds than slow‐wilting soybean pods, but slow‐wilting soybean seeds had a significantly higher mass and diameter than fast‐wilting soybean seeds, suggesting a potential trade‐off in fitness which may impact production quality (Kumagai, Yabiku, and Hasegawa 2022). While there was no significant difference in the number of seeds per pod or seed mass across drought and herbivory treatments, seeds from soybean plants that experienced a combination of drought and herbivory had a larger diameter than seeds that experienced only drought or control plants.

In the past, slow‐wilting soybean plants produced significantly more pods than fast‐wilting plants, but when faced with compounding stress from drought and herbivory, fast‐wilting soybean plants tend to overcompensate in terms of growth and yield: growing taller with more leaves and producing more pods (Ayala et al. 2024; Gautam, Shafi, and Kariyat 2024). This may be indicative of a delayed effect that despite the overcompensation by fast‐wilting soybean plants in the production of pods, the pods and seeds produced may be of lower quality compared to slow‐wilting soybean plants (Peschiutta et al. 2020).

As an additional measure of fitness, we also examined germination rates and found no significant difference in the percentage of soybean plants that germinated between soybean wilting speeds or across drought and herbivory treatments. There was no significant difference in the number of seeds that germinated per date between soybean wilting speeds or across drought and herbivory treatments. While this aspect of fitness was not affected by wilting speed or treatment, there may be lasting effects in the plants in terms of yield or pod quality later in their life history, a delayed transgenerational effect that we are currently exploring (Peschiutta et al. 2020).

Overall, there was a significant genotypic effect throughout the study. When observing plant growth traits and chlorophyll, all the genotypes were significantly different from each other. Similarly, the genotypes were significantly different for physiological traits including net photosynthesis, stomatal conductance, transpiration rate, and intracellular CO_2_. Despite the significant diversity across plant growth and physiological traits, there was no significant difference in FAW herbivory across the genotypes, including FAW leaf damage or FAW percent mass gain. However, there was a significant difference in SBL leaf damage across genotypes, but no significant difference in SBL mass gain. For yield traits, there was a significant difference across all soybean genotypes in their pod number, pod mass, number of seeds per pod, seed mass, and seed diameter. Finally, there was a significant difference in percent germination as well as germination by date across all genotypes. Collectively, the abundance of diversity in soybean genotypes for these traits, while more or less consistent with wilting speed, demonstrates the complexity of the genes associated with wilting speed and may assist in further identifying the interactions between WUE traits and herbivore resistance for cultivar development (Sinclair et al. 2010; Carmona, Lajeunesse, and Johnson 2011; Grinnan, Carter, and Johnson 2013).

Our initial hypothesis was ultimately contradicted as the combination of drought and insect herbivory caused the soybean plants to overcompensate and grow taller, produce more leaves, and produce more pods. Additionally, we expected that the soybean‐dominant herbivore, SBL, would be able to overcome the plant's defenses and gain more mass compared to the generalist herbivore, FAW (Chen et al. 2018; Kenis et al. 2022). However, contrary to our expectations, FAW continues to feed at a significantly higher rate than SBL. We also predicted that slower‐wilting soybean genotypes would better defend against insect herbivory. While it was not statistically significant, we did observe FAW feed more on slow‐wilting compared to fast‐wilting leaf tissue and gain less mass when feeding on slow‐wilting leaf tissue when compared to fast‐wilting leaf tissue (Ayala et al. 2024). There is evidence to suggest that the FAW prefers slow‐wilting soybeans because they exhibit less stress, demonstrated by the higher quality of their seeds (Mubayiwa et al. 2023). However, they may struggle to gain mass on slow‐wilting soybean plants due to the higher levels of nitrogen fixation and therefore photosynthesis, potentially allowing the plants to have higher levels of resistance (Zvereva and Kozlov 2006; Bellaloui et al. 2013).

Future studies should focus on examining the continued life history of both FAW and SBL following exposure to fast‐wilting and slow‐wilting soybean genotypes that experienced drought and herbivory treatment. This may give further insight into the true impact of the combination of abiotic and biotic stressors on pest growth and development. The combination of 12 genotypes and extensive trait examination from morphophysiology to fitness on fast‐wilting and slow‐wilting soybean genotypes under stress in this experiment provides an excellent starting point to dig deeper into mechanisms and teasing out individual genotypic effects—for breeding and cultivar development. Further understanding of how the combination of different wilting speeds and drought conditions influences pest resistance in the field is necessary to produce soybeans that maximize yield while deterring insect pests.

## Conflicts of Interest

The authors declare no conflicts of interest.

## Supporting information


Data S1.

## Data Availability

Raw data collected from this experiment are available at Jessica PEI raw data on U. Arkansas One Drive.
