# Supplementary material for: Pulsating Drought and Insect Herbivory Cause Differential Effects on Soybean ( Glycine max ) Genotypes That Vary in Canopy Wilting Speed
Source: Plant Environ Interact. 2025 Jan 30;6(1):e70028. doi: 10.1002/pei3.70028 (PMC11781298; doi:10.1002/pei3.70028)
Supplement: Supplementary file 1 — Data S1. [file PEI3-6-e70028-s001.docx]

Supplementary files:

A)
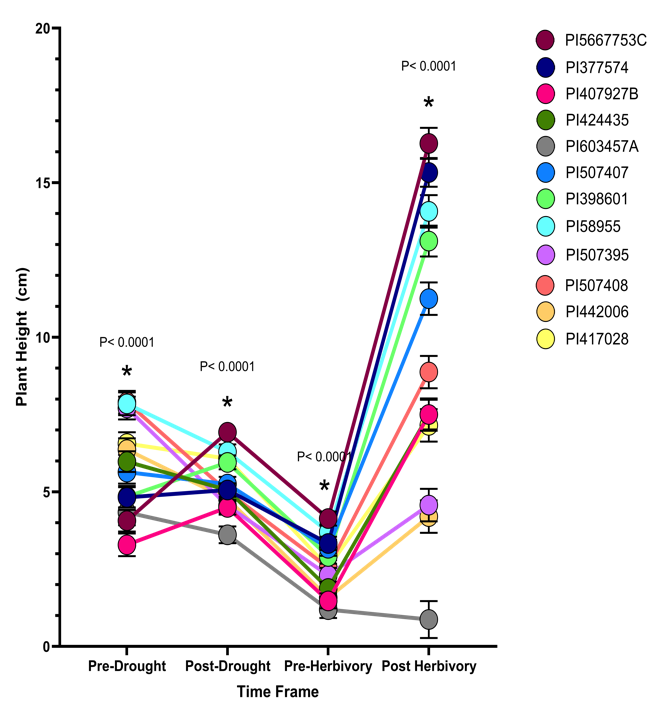
B)
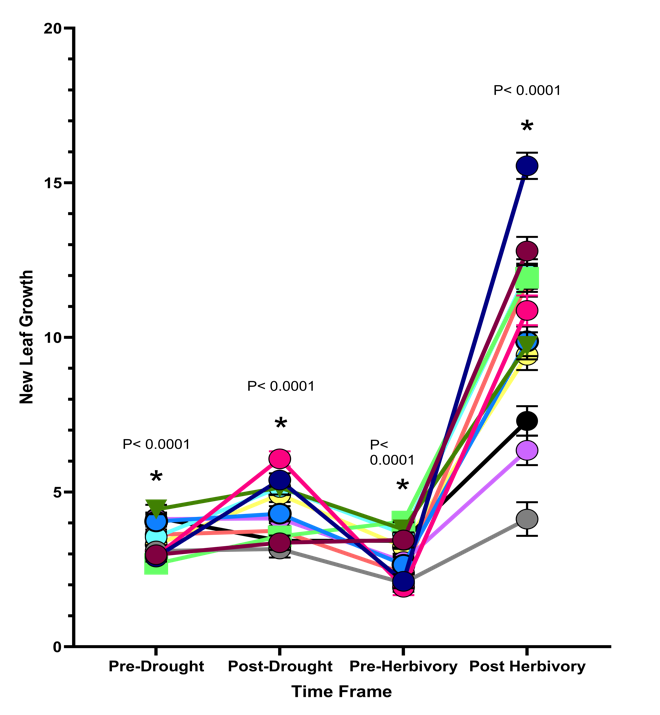


C)
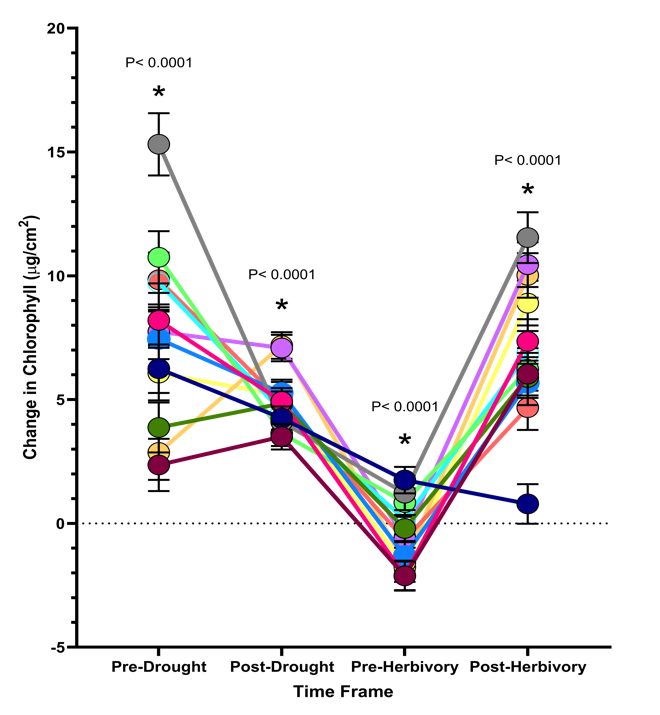


**Figure 1.** Changes in Plant Traits Over the Course of the Study. A) Change in mean plant height growth over the course of the study across soybean genotypes (Kruskal-Wallis; p <0.0001). B) Change in mean number of leaves gained over the course of the study across soybean genotypes (Kruskal-Wallis; p <0.0001). C) Change in mean chlorophyll content over the course of the study across soybean genotypes (Kruskal-Wallis; p <0.0001). Asterisks denote significant differences in mean mass at the 5 % level of significance, and data are presented as mean ± SE (standard error).

A)
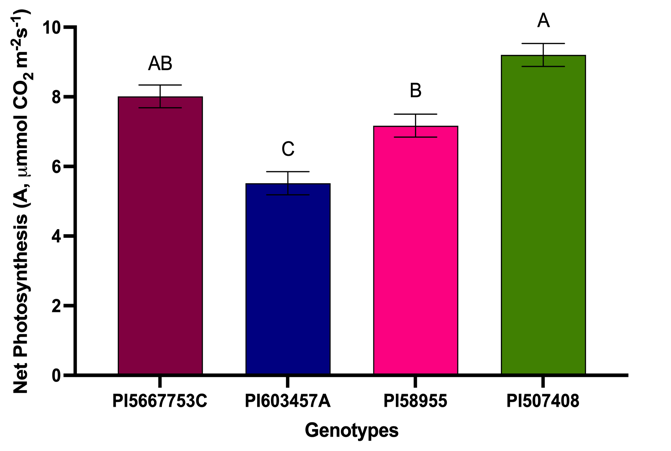
B)
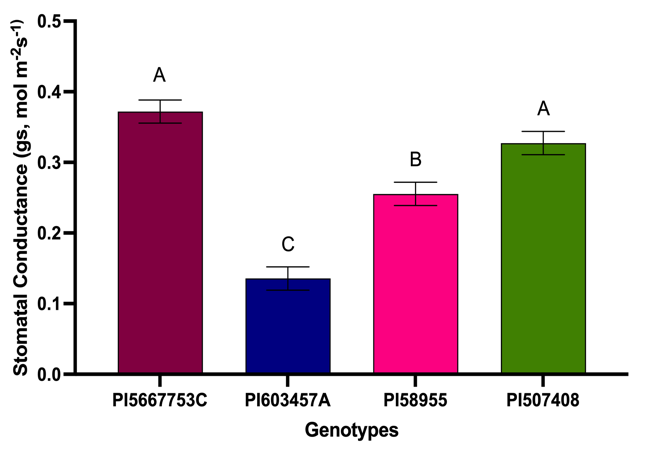


C)
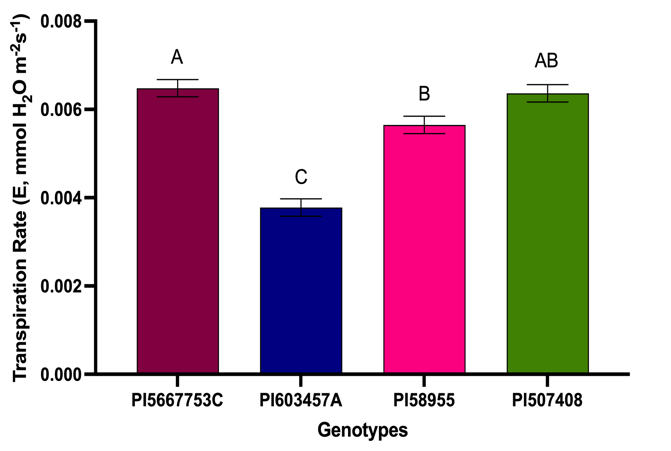
D)
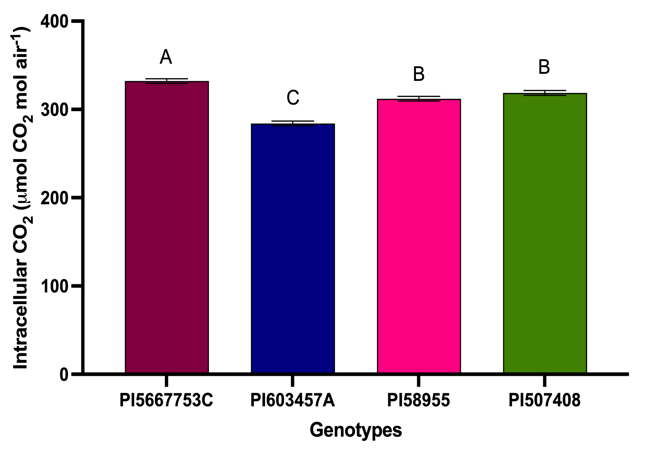


**Figure 2.** LICOR. A) Mean net photosynthesis across soybean genotypes (ANOVA; p <0.0001). B) Mean stomatal conductance across soybean genotypes (ANOVA; p <0.0001). C) Mean transpiration rate across soybean genotypes (ANOVA; p <0.0001). D) Mean intracellular CO_2_ across soybean genotypes (ANOVA; p <0.0001). The measurements were taken from 6 plants from each genotype. Different letters denote significant differences in mean mass at the 5 % level of significance, and data are presented as mean ± SE (standard error).

A)
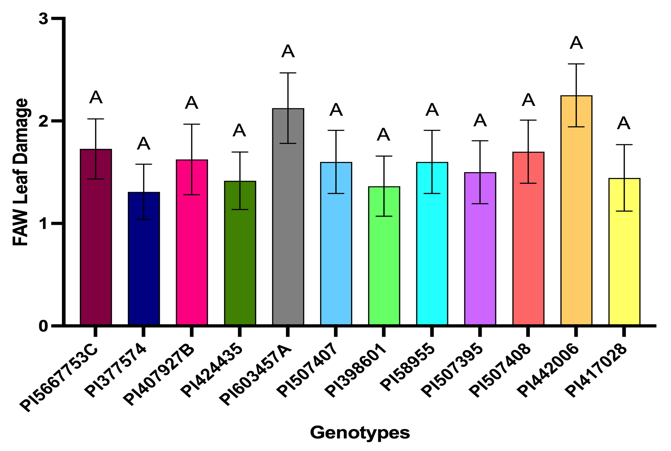
B)
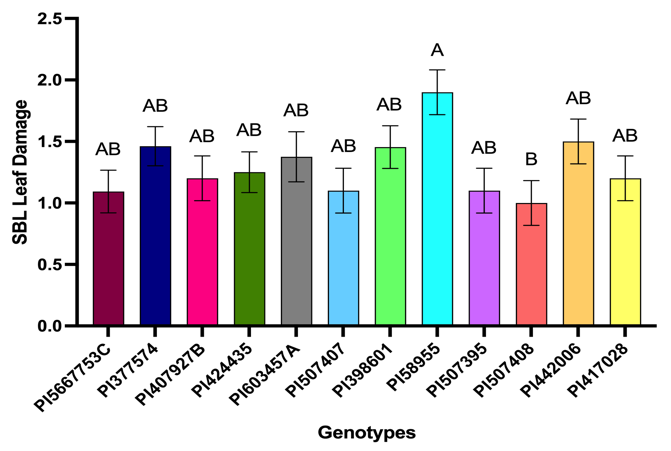
C)
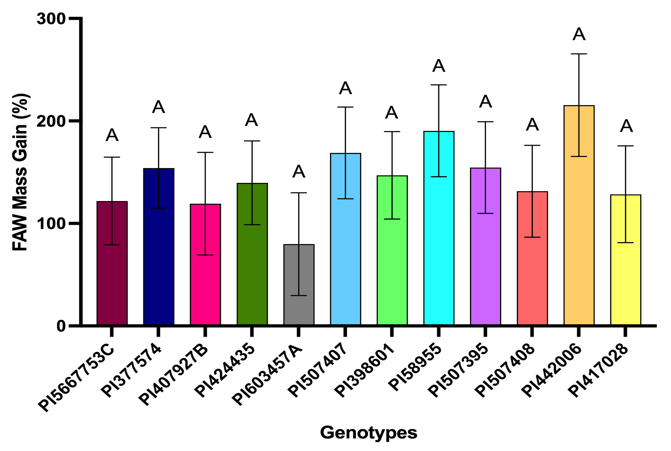
D)
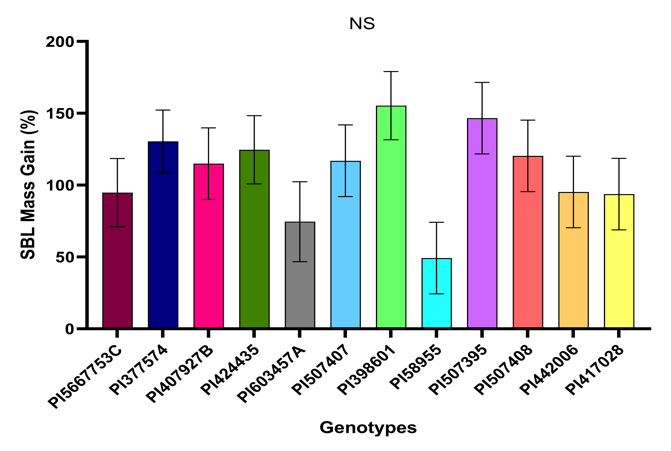


**Figure 3.** Herbivory Traits. A) Mean leaf damage inflicted by FAW across soybean genotypes (ANOVA; p= 0.6424). B) Mean leaf damage inflicted by SBL across soybean genotypes (ANOVA; p= 0.0415). C) Mean percent mass gained by FAW across soybean genotypes (ANOVA; p= 0.8775). D) Mean percent mass gained by SBL across soybean genotypes (ANOVA; p= 0.1555). Different letters denote significant differences in mean mass at the 5 % level of significance, and data are presented as mean ± SE (standard error).

A)
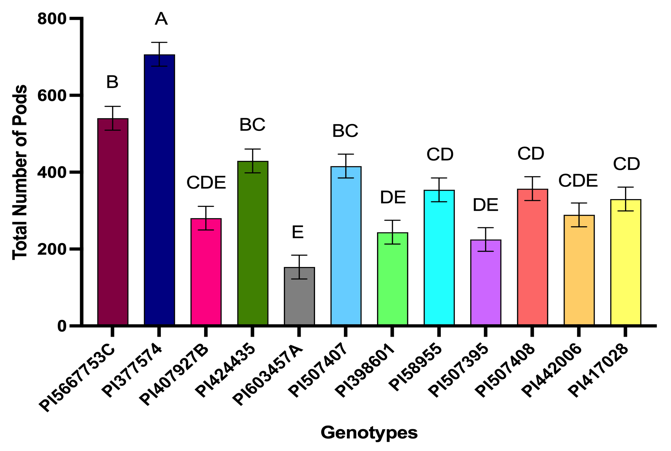
B)
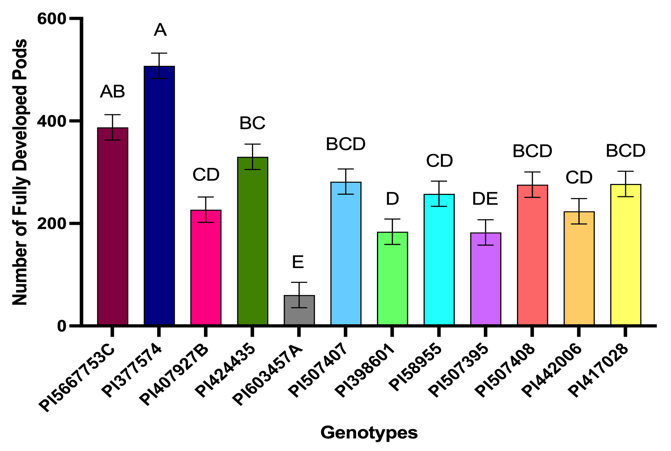


C)
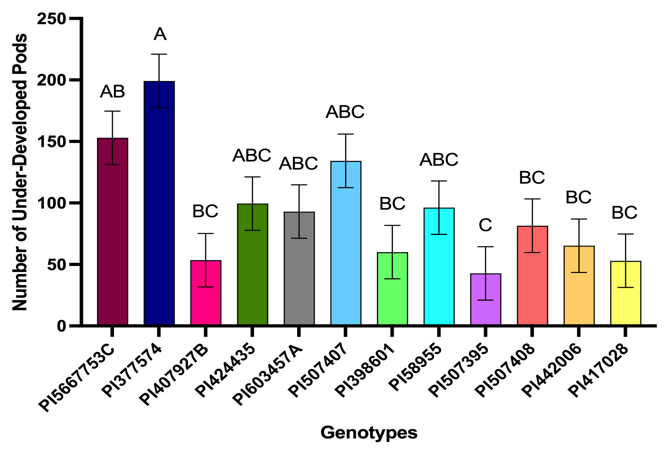
D)
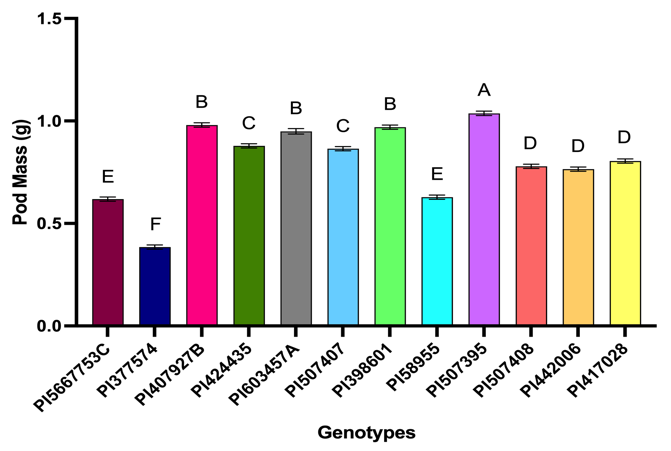


**Figure 4**. Yield Traits: Pods. A) Mean number of total pods across soybean genotypes (ANOVA; p<0.0001). B) Mean number of fully developed soybean pods across soybean genotypes (ANOVA; p<0.0001). C) Mean number of underdeveloped soybean pods across soybean genotypes (ANOVA; p= 0.0002). D) Mean soybean pod mass across soybean genotypes (ANOVA; p<0.0001). Different letters denote significant differences in mean mass at the 5 % level of significance, and data are presented as mean ± SE (standard error).

A)
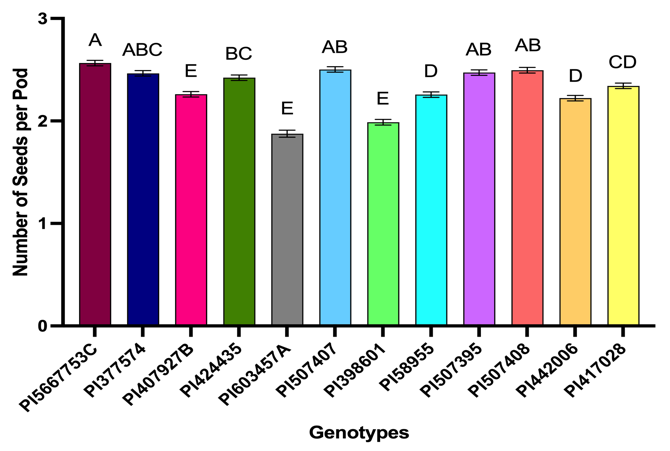
B)
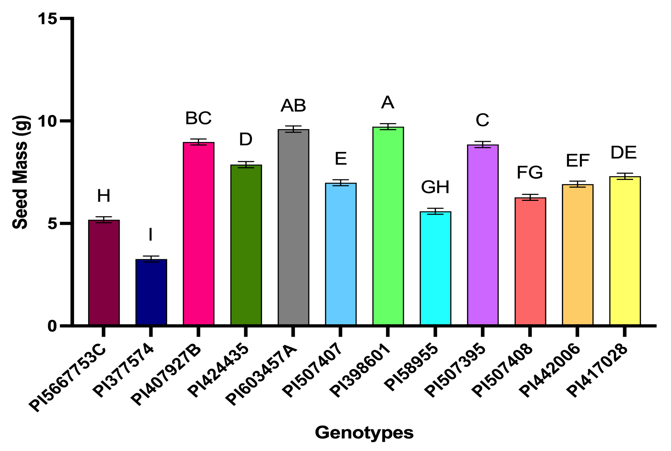


C)
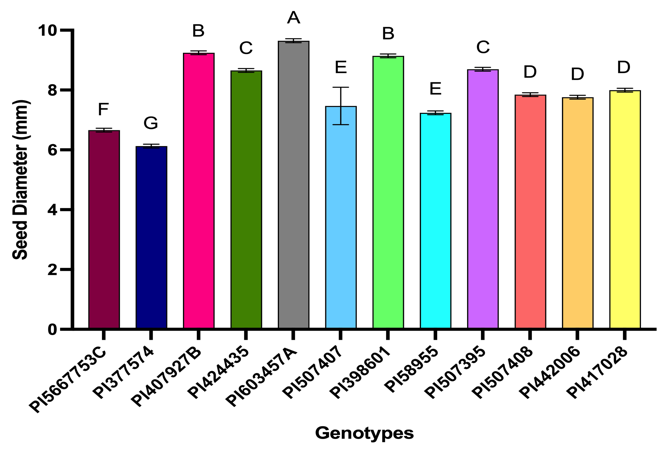


**Figure 5**. Yield Traits: Seeds. A) Mean number of seeds per pod across soybean genotypes (ANOVA; p <0.0001). B) Mean seed mass across soybean genotypes (ANOVA; p <0.0001). C) Mean seed diameter across soybean genotypes (ANOVA; p<0.0001). Different letters denote significant differences in mean mass at the 5 % level of significance, and data are presented as mean ± SE (standard error).

A)
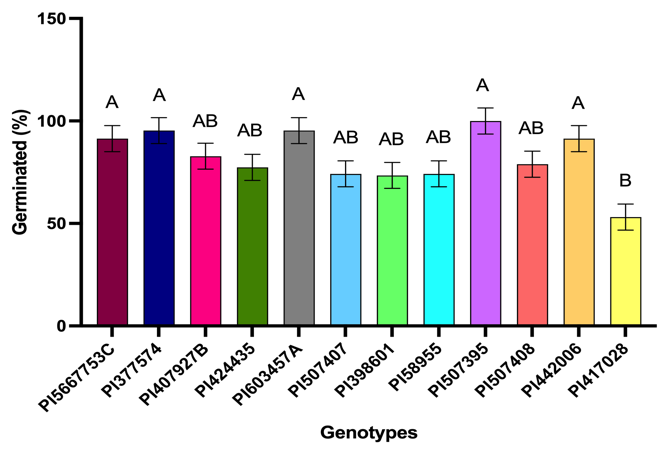
B)
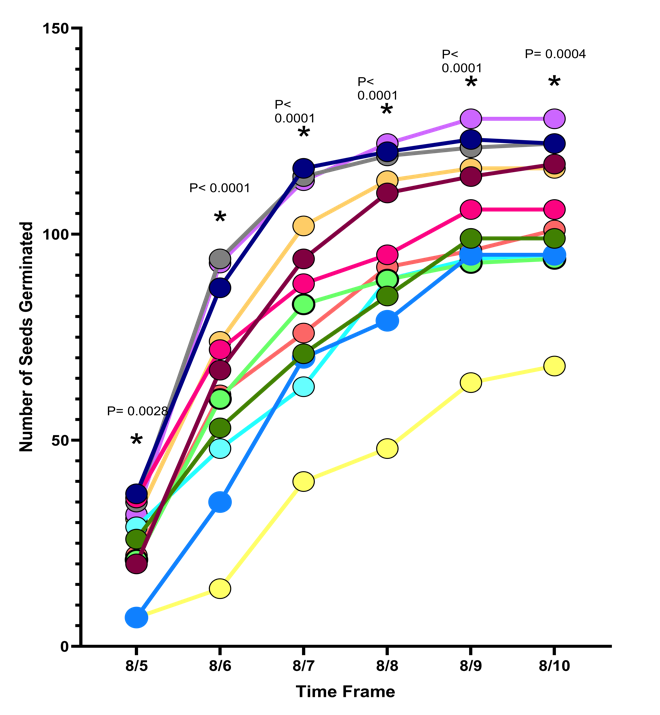


**Figure 6.** Germination Study. A) Mean percentage of seeds that germinated across soybean genotypes (ANOVA; p=0.0004). Different letters denote significant differences in mean mass at the 5 % level of significance, and data are presented as mean ± SE (standard error). B) Total number of soybean seeds that germinated in each day across 6 days (ANOVA; p=0.0005). Asterisks denote significant differences in mean mass at the 5 % level of significance, and data are presented as mean ± SE (standard error).

**Table 1**. ANOVA for the effect of soybean genotype on plant and herbivory traits.

| Trait | Source of Variation | df | SS | F | P |
| --- | --- | --- | --- | --- | --- |
|  |  |  |  |  |  |
| LICOR | Genotype X Net Photosynthesis  Genotype X Stomatal Conductance  Genotype X Transpiration Rate  Genotype X Intracellular CO_2_ | 3  3  3  3 | 514.56377  2.2909229  0.00033508  87294.627 | 21.9518  39.0237  40.1377  55.5295 | **<0.0001**  **<0.0001**  **<0.0001**  **<0.0001** |
|  |  |  |  |  |  |
| Herbivory: Leaf Damage | Genotype X FAW Leaf Damage  Genotype X SBL Leaf Damage | 11  11 | 8.3062743  7.0558671 | 0.7973  1.9385 | 0.6424  **0.0415** |
|  |  |  |  |  |  |
| Herbivory: Mass Gain (%)  Yield: Pods  Yield: Seeds  Germination Study | Genotype X FAW Mass Gain (%)  Genotype X SBL Mass Gain (%)  Genotype X Total Pod Number  Genotype X Fully Developed Pods  Genotype X Underdeveloped Pods  Genotype X Pod Mass  Genotype X Seeds per Pod  Genotype X Seed Mass  Genotype X Seed Diameter  Genotype X Percent Germination | 11  11  11  11  11  11  11  11  11  11 | 117519.37  99732.52  989969.23  548362.5  97202.729  151.1847  168.84949  493.11352  1502.1433  7657.8776 | 0.5324  1.4631  23.5439  20.3741  4.6804  315.3676  53.1355  171.607  290.2281  4.3168 | 0.8775  0.1555  **<0.0001**  **<0.0001**  **0.0002**  **<0.0001**  **<0.0001**  **<0.0001**  **<0.0001**  **0.0004** |
|  |  |  |  |  |  |
